# Altered Neocortical Dynamics in a Mouse Model of Williams–Beuren Syndrome

**DOI:** 10.1007/s12035-019-01732-4

**Published:** 2019-08-30

**Authors:** Miguel Dasilva, Alvaro Navarro-Guzman, Paula Ortiz-Romero, Alessandra Camassa, Alberto Muñoz-Cespedes, Victoria Campuzano, Maria V. Sanchez-Vives

**Affiliations:** 1 Institut d’Investigacions Biomèdiques August Pi i Sunyer (IDIBAPS), Barcelona, Spain; 2 Departament de Ciències Experimentals i de la Salut, Universitat Pompeu Fabra, Barcelona, Spain; 3 Laboratorio Cajal de Circuitos Corticales (CTB), Universidad Politécnica de Madrid, Madrid, Spain; 4 Depatamento de Biología Celular, Universidad Complutense, Madrid, Spain; 5 Centro de Investigación Biomédica en Red de Enfermedades Raras (CIBERER), ISCIII, Barcelona, Spain; 6 Institució Catalana de Recerca i Estudis Avançats (ICREA), Barcelona, Spain

**Keywords:** Up states, Slow oscillations, Synchronization, Cerebral cortex, Cortical dynamics, Cognitive deficit

## Abstract

Williams–Beuren syndrome (WBS) is a rare neurodevelopmental disorder characterized by moderate intellectual disability and learning difficulties alongside behavioral abnormalities such as hypersociability. Several structural and functional brain alterations are characteristic of this syndrome, as well as disturbed sleep and sleeping patterns. However, the detailed physiological mechanisms underlying WBS are mostly unknown. Here, we characterized the cortical dynamics in a mouse model of WBS previously reported to replicate most of the behavioral alterations described in humans. We recorded the laminar local field potential generated in the frontal cortex during deep anesthesia and characterized the properties of the emergent slow oscillation activity. Moreover, we performed micro-electrocorticogram recordings using multielectrode arrays covering the cortical surface of one hemisphere. We found significant differences between the cortical emergent activity and functional connectivity between wild-type mice and WBS model mice. Slow oscillations displayed Up states with diminished firing rate and lower high-frequency content in the gamma range. Lower firing rates were also recorded in the awake WBS animals while performing a marble burying task and could be associated with the decreased spine density and thus synaptic connectivity in this cortical area. We also found an overall increase in functional connectivity between brain areas, reflected in lower clustering and abnormally high integration, especially in the gamma range. These results expand previous findings in humans, suggesting that the cognitive deficits characterizing WBS might be associated with reduced excitability, plus an imbalance in the capacity to functionally integrate and segregate information.

## Introduction

Williams–Beuren syndrome (WBS) is a rare neurodevelopmental disorder that results from the heterozygous deletion of 26–28 contiguous genes at chromosome 7q11.23 and is characterized by mild to moderate intellectual disability, learning difficulties, and hypersociability [1]. Brain anatomy and structure are also affected in WBS at the macroscopic and microscopic levels. Abnormalities in the structure of the amygdala, hippocampus, and cerebral cortex have been described and related to the neurocognitive profile [2]. Anomalies in the cytoarchitecture of the cerebral cortex, specifically columnar orientation and cell and dendritic density, have been reported [3–6]. Sleep structure and efficiency have also been described as altered in WBS. Specifically, sleep cycles are fragmented and disorganized, REM sleep is decreased, and the frequency content of NREM sleep is unbalanced [7, 8]. In addition, alterations in functional connectivity have been described during resting states and natural sleep in individuals suffering from WBS [9–11].

Slow oscillations (SO) are an intrinsic emergent phenomenon of the cortical network that surge spontaneously during NREM sleep and anesthesia [12–15]. SO are characterized by the alternation of periods of high-firing and high-frequency content (called Up states), which resemble activity during wakefulness, and Down states, where neuronal activity is almost silent [12, 16, 17]. Different SO parameters such as the magnitude of neuronal firing, the periodicity of the oscillatory cycle, and the duration of Up and Down states or the high-frequency content, in addition to functional connectivity, have been recently demonstrated to be altered in different mouse models of autism, aging, and intellectual disability [18–24]. SO are thus a highly revealing activity of the cerebral cortex and their properties can be highly indicative of underlying abnormalities in the functional and anatomical domain.

The frontal cortex subserves higher-order cognitive functions and social behavior and has been related to normal sleep physiology [25], parameters that are affected in WBS. Here, we have focused on the analysis of different properties of SO activity in the frontal cortex of anesthetized mice in order to study the intrinsic functional alterations of the cortical brain activity underlying the neurocognitive impairment and sleep disturbances present in WBS. We have also explored an anatomical property of these neurons, carrying out a quantification of dendritic spines in the frontal area, and the cortical activity during the realization of cognitive tasks in the awake state. To this end, we used a mouse model of WBS recently reported to replicate most of the cognitive and behavioral phenotype present in human individuals affected by this condition [26]. We found that the duration of Down states is more variable in these animals, associated with a reduced firing rate alongside a decrement in the power of high-frequency content in the gamma range; features that were also apparent in the awake animal during the realization of a cognitive task. We also found a decrease in spine length and density and alterations in functional connectivity. These alterations may offer further insights into the cortical alterations observed in WBS.

## Methods

### Animals

All procedures were approved by the Ethics Committee at the Hospital Clinic of Barcelona and were carried out to the standards laid down in Spanish regulatory laws (BOE-A-2013-6271) and European Communities Directive (2010/63/EU). Recordings were performed in adult (3–4 months old) male mice carrying a heterozygous deletion from *Gtf2i* to *Fkbp6*, obtained as previously described [26] and maintained on at least 97% C57BL/6J background. WBS mutant mice were crossed with Thy1-YFP transgenic mice (B6.Cg-Tg(Thy1-YFPH)2Jrs/J, Jackson Laboratory) to label pyramidal neurons. All animals were bred in-house at the University of Barcelona and kept under 12-h light/dark cycle with food and water *ad libitum*.

### Surgical Procedures and Recording

Anesthesia was induced with an intraperitoneal injection of ketamine (100 mg/kg) and medetomidine (1.3 mg/kg) and maintained by constant administration of subcutaneous ketamine (37 mg/kg/h). Atropine (0.3 mg/kg), methylprednisolone (30 mg/kg), and mannitol (0.5 g/kg) were administered subcutaneously to avoid respiratory secretions and edema. Once in the surgical plane of anesthesia, a tracheotomy was performed to aid breathing and the animal was placed on a stereotaxic frame (SR-6M, Narishige, Japan) with body temperature constantly monitored and kept at 37 °C by means of a thermal blanket (RWD Life Science, China). For experiments in the frontal cortex, a craniotomy and durotomy were performed over the left or right hemisphere at 2.6 mm relative to the bregma and 1.3 mm relative to the midline.

### Electrophysiological Recordings in the Anesthetized Animal

Extracellular local field potential (LFP) activity was recorded by means of 16-channel multielectrode penetrating probes (100 μm spacing, E16–100-S1-L6, Atlas Neuroengineering, Leuven, Belgium) spanning the entire depth of the cortex. To ensure consistency across experiments, probes were lowered orthogonally to the cortex under visual inspection until the last recording point was aligned with the surface of the brain. For experiments aimed at studying wave propagation and functional connectivity, a wide craniotomy and durotomy were performed over the left or right hemisphere from − 3.0 mm to + 3.0 mm relative to the bregma and + 3.0 mm relative to the midline. Extracellular micro-electrocorticogram (micro-ECoG) activity was recorded subdurally using 32-channel multielectrode arrays (550 μm spacing) covering a large part of the surface of one hemisphere. In both types of experiments, the signal was amplified (Multichannel Systems, GmbH), digitized at 5 kHz, and fed into a computer via a digitizer interface (CED 1401 and Spike2 software, Cambridge Electronic Design (CED), UK).

### Electrophysiological Recordings in the Behaving Animal

Animals were anesthetized with isoflurane and bilaterally implanted in the frontal cortex (anteroposterior 2.6 mm from the bregma, mediolateral 1.3 mm from the midline) with bipolar electrodes made of Teflon™-coated tungsten wire (50 μm thick, Advent Research Materials) in order to record local field potential activity (LFP). Electromyographic activity (EMG) was also acquired using two teflon-coated tungsten wires (50 μm thick, Advent Research Materials) implanted in the neck muscles. Electrodes were affixed in all cases to the skull by means of dental cement and steel screws and soldered to an interface plastic socket (RS Amidata) connected to the recording system. LFP and EMG activity were amplified by 100 (PGA, Multichannel Systems), digitized at 10 KHz, and fed into a computer via a digitizer interface (CED and Spike2 software, CED).

### Propagation

From the extracellular recordings of slow-wave activity under different conditions, the multiunit activity (MUA) was estimated as the high-frequency component (200–1500 Hz) of the Fourier transform of the LFP [27] (see below). Under the slow oscillatory regime, the Up and Down states were detected using a threshold on the logarithmically scaled MUA of each recording electrode. The waves were reconstructed by merging the transitions belonging to the same wave (i.e., the ones occurring in the majority of the channels in a reasonable time interval) and expressed as time lags with respect to each mean wave time. The spatiotemporal profile of propagation of the waves was computed by interpolating the time lags *T*(*x*, *y*) using a thin-plate spline technique without smoothing, as described by Capone et al. [28] The gradient of the interpolated time lags was computed and used to estimate the absolute speed *V*(*x*, *y*) as:

$$ V\left(x,y\right)=\frac{1}{\sqrt{{\left(\frac{\partial T\left(x,y\right)}{\mathrm{\partial x}}\right)}^2+{\left(\frac{\partial T\left(x,y\right)}{\mathrm{\partial y}}\right)}^2}} $$

The projection of the TimeLagMatrix on the first two principal component spaces was used to compute a measure of complexity based on the entropy of wavefronts’ activation [29].

### Functional Connectivity, Modularity, and Path Length Analysis

For the wideband analysis, recorded LFPs were downsampled and pairwise correlated using Pearson’s correlation coefficient. To study the functional connectivity at specific frequency bands, LFPs were also zero-phased bandpass filtered with a Butterworth filter. The resulting 32 × 32 matrices of each individual were used as input for the Louvain method for community detection and average shortest path analysis adapted from [30]. For the shortest path calculations, a conversion between correlation strength (*W*_*ij*_) and distance (*D*_*ij*_) was performed as:

$$ {D}_{ij}=\frac{1}{W_{ij}} $$

Functional complexity based on connectivity matrices was measured following Zamora-Lopez et al. [31].

### Behavior: Marble-Burying Test

The marble burying test was chosen based on results previously obtained in the WBS model [32–34]. An open test arena (26 × 26 cm) was filled with bedding to a depth of 5 cm, and 20 glass marbles were placed on the surface following a regular pattern (5 rows of 4 marbles). During each session, a single mouse (WBS or wild-type (WT)) was connected to the recording system and left to freely explore the testing arena for 20 min while LFP activity was recorded. After an initial exploratory time (usually between 5 and 10 min), animals began to bury the marbles until most of them were covered with bedding ( >2/3 marble covered). The recorded LFP activity of WBS and WT mice during the execution of the task was later compared.

### Histology

At the end of electrophysiological recording experiments, the aorta was perfused with saline solution followed by paraformaldehyde (4%) in 0.1 M phosphate buffer pH 7.4 (PB). Brains were then removed, postfixed, and cryoprotected in 30% sucrose in PB until they sank and then frozen in dry ice. Coronal brain sections (50 μm) were taken with a freezing sliding microtome (Microm HM 450, Microm International, Germany). Sections were mounted on gelatin-coated histological slides and Nissl-stained with Toluidine blue. Sections were then dehydrated in ethanol, cleared in xylene, and coverslipped with DePeX. Sections were photographed at ×4 with a DP70 camera attached to a BX51 light microscope (Olympus). The thickness of cortical layers and distance to the cortical pial surface of the limits between cortical layers, identified by changes in cell size and packing density, were measured in at least five sections of the frontal association cortical area in each WT and WBS animal. As brain tissue shrinks during processing, mean values of lineal measurements were corrected for shrinkage by dividing them by a factor of 0.71, estimated by measuring the surface area of the sections using Adobe Photoshop before and after tissue processing.

### Dendrite Imaging

We obtained 1024 × 1024 pixel confocal fluorescent image stacks from coronal tissue sections of 150 μm using a TCS SP2 LEICA confocal microscope. For spine quantification, an HC PL FLUOTAR 63× (zoom 5) oil-immersion objective was used. Apical proximal dendrites (secondary apical dendrites, 50–150 μm from soma) were selected for the analysis of spine density. The number of spines was counted using ImageJ Cell Counter plugging 15–30 μm dendritic segments of randomly selected neurons. Spine counts included all types of dendritic protrusions. Spines located on the top or bottom surfaces of the dendrites were not counted; thus, the total number of spines was underestimated in all cases. Six animals were analyzed per genotype and 6–12 dendritic segments were analyzed per animal. Spine density was calculated by dividing the total spine count by the length of dendrite analyzed. Spine length was measured from the base of the spine neck to the end of the head of the spine. We analyzed 75–200 spines/animal with a total number of 518 (WBS) and 689 (WT) spines analyzed.

### Data Analysis and Statistics

The detection of Up and Down states was based on LFP deflections, gamma rhythm, and neuronal firing as previously described [15]. After detection, the average duration of Up and Down states was calculated and the frequency of the SO was estimated as the inversion of the complete Up-to-Down cycle duration. Population firing rate was estimated based on the power spectrum density of the SO signal between 200 and 1500 Hz. The normalized MUA spectrum provides a solid estimate of the population firing rate, given that normalized Fourier components at high frequencies have densities proportional to the spiking activity of the involved neurons, while also allowing removal of the linear response associated with electrode impedance [35]. The high-frequency content during Up states was calculated after performing a Welch power spectrum density analysis.

Statistical significances were assessed by the Mann–Whitney *U* test. A Kolmogorov–Smirnov test was performed to study differences in the correlation distribution of the functional connectivity study. Data are presented as mean ± SEM. All data analysis and statistics were implemented in MATLAB (MathWorks).

## Results

**Fig. 1:**
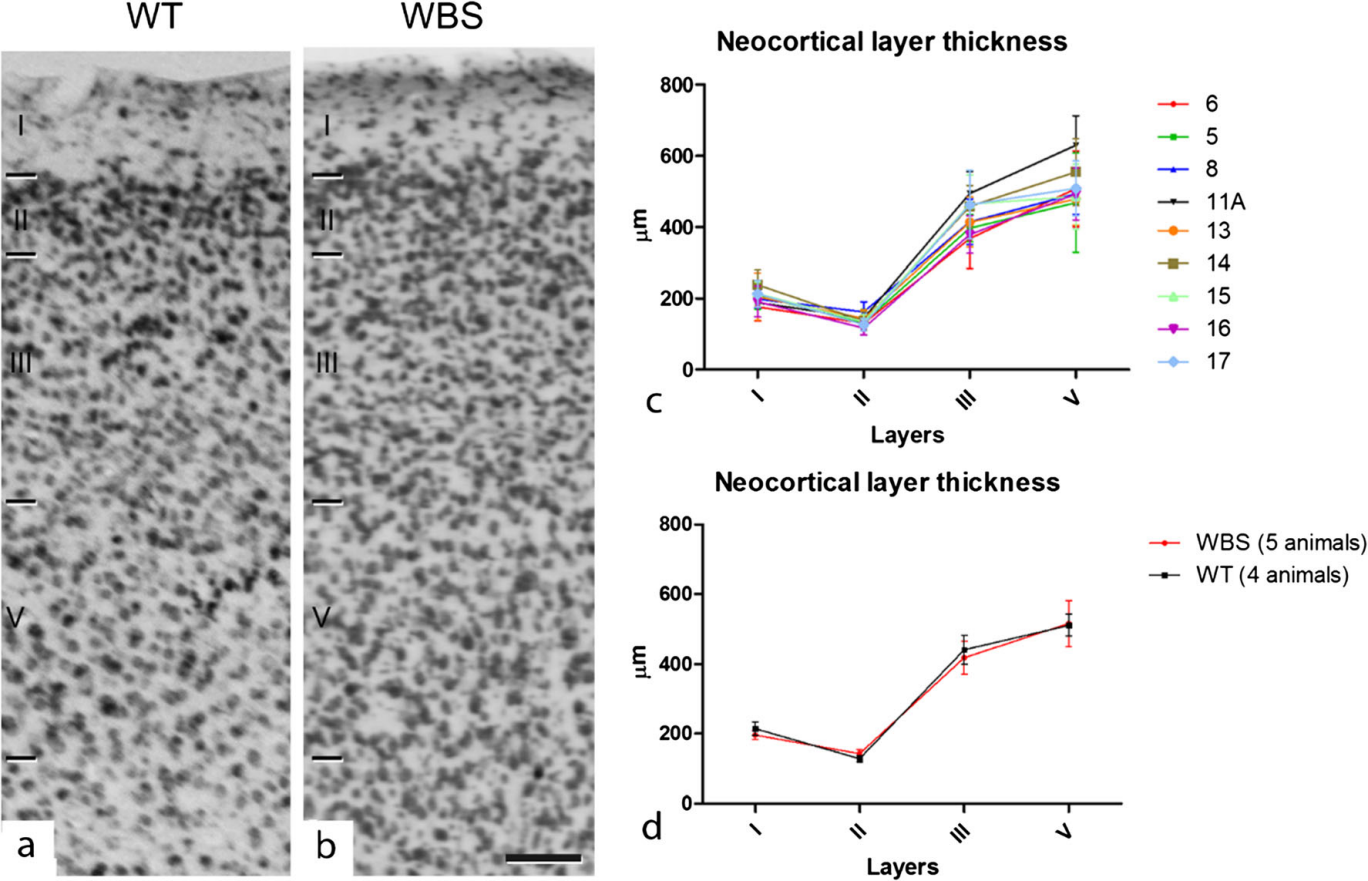
Cortical lamination of frontal association area in WT and WBS mice. Photomicrographs from Nissl-stained sections through the frontal association cortex of WT (**a**) and WBS (**b**) mice showing the limits between cortical layers. Scale bar indicates 95 μm. **c**, **d** Line graphs showing the lack of differences in layer thickness between WT and WBS mice

Anatomical differences have been reported in WBS such as reduced brain size in humans [36], reduced lateral ventricle, and increased neuronal density in the somatosensory cortex of a double heterozygous (D/P) and partial deletion (PD) mice [37]. Since our objective was to record from the cortical layers of the frontal association area, we first carried out an anatomical study to estimate possible differences in the thickness of the individual layers and also the global thickness of the cortex both in WT and WBS mice. The frontal cortex of 10 mice (WT = 4; WBS = 6) was studied with Nissl (see “Methods”; Fig. 1a, b) and the cortical layers identified and measured (Fig. 1c, b). No significant differences were detected between the thickness of cortical layers of the frontal cortex between WT and WBS. We also measured the depth from the pial surface of the limits between layers, according to previous studies indicating the lack of a well-developed layer IV in rodents [38, 39].

**Fig. 2:**
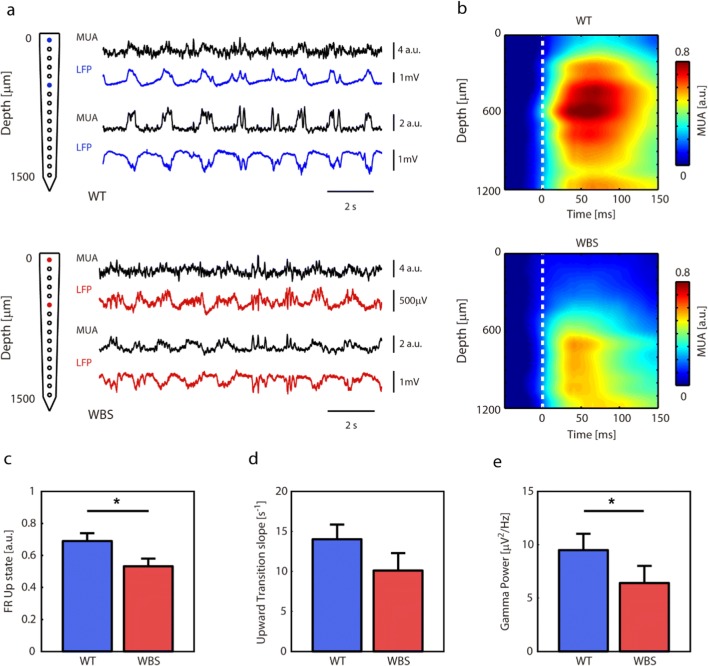
In-depth recordings from frontal association area in WT and WBS mice. (**a**) Electrophysiological recordings from WT (top) and WBS (bottom) representing slow oscillatory activity (MUA, LFP) at two depths (0 and 500 μm). (**b**) Grand average of MUA with respect to depth (*N*_WT_ = 9, *N*_WBS_ = 8). (**c**) Frequency of occurrence of the Up states in WT and WBS mice (*p* = 0.0360, Mann–Whitney *U* test). (**d**) Down to Up slope (indicating speed of recruitment of the local network) in WT and WBS mice (*p* = 0.1672, Mann–Whitney *U* test). (**e**) Gamma power in the power spectrum density (32–100 Hz) (*p* = 0.0464, Mann–Whitney *U* test)

We then recorded the spontaneous LFP activity generated in the frontal cortex of 9 WBS mice and 8 WT littermates under ketamine anesthesia. As previously reported [15, 19, 22], under these conditions SO emerge, and cortical activity alternates between periods of neuronal firing (or Up states) interspersed with relatively silent periods (or Down states) (Fig. 2a). SO parameters such as Up or Down state duration, population firing rate during Up and Down states, or high-frequency content, have been shown to be affected in murine models of aging and mental disability [15, 18, 19, 22, 24]. With the objective of investigating whether this emergent activity would be altered in the WBS model, we recorded the activity generated in the cortico-frontal association area using multielectrode arrays (Fig. 2a). We estimated the population firing rate as the normalized MUA spectrum, given that normalized Fourier components at high frequencies have densities proportional to the spiking activity of the involved neurons [35]. When the population firing rate was analyzed with respect to cortical depth (Fig. 2b), we found that the highest firing rates corresponded to 500–800 μm (layers 3 and 5, Fig. 1; note that there is no layer 4 in the frontal cortex). We thus quantified different parameters of the SO for the four channels corresponding to these depths. The quantified parameters of the SO and their statistics are displayed for WT and WBS in Table 1. We found that the duration of Up and Down states was not different between WBS and WT mice, which was reflected in a similar SO frequency. However, the regularity of  Down states (as measured by the coefficient of variation of Down state durations) was lower, and the SO cycle less regular, in WBS animals. We also found that the firing rate during Up states was significantly lower in WBS mice (Fig. 2c). The slope of the transition from Down to Up states (Fig. 2d), which gives a measure of the local network recruitment towards the Up state, follows a similar (though non-significant) trend.

**Table 1:** Parameters of the cortical activity during slow oscillations in frontal association area in WT and WBS mice. Mean ± SEM. Mann–Whitney *U* test and *p* value (* *p*=<0.05)

	WT	WBS	*p* value
Up duration (s)	0.39 ± 0.01	0.4 ± 0.02	*p* = 0.606
Down duration (s)	0.82 ± 0.07	0.89 ± 0.09	*p* = 0.606
SO frequency (Hz)	0.85 ± 0.08	0.80 ± 0.10	*p* = 0.673
CV Up duration (a.u.)	0.51 ± 0.04	0.63 ± 0.06	*p* = 0.114
CV Down duration (a.u.)	0.49 ± 0.03	0.6 ± 0.04	*p* = 0.046*
CV SO frequency (a.u.)	0.37 ± 0.02	0.47 ± 0.04	*p* = 0.036*
Alpha PSD (μV^2^/Hz)	200 ± 30	140 ± 30	*p* = 0.139
Beta PSD (μV^2^/Hz)	49 ± 7	36 ± 8	*p* = 0.167
Gamma PSD (μV^2^/Hz)	9 ± 2	6 ± 2	*p* = 0.046*
Up FR (a.u.)	0.69 ± 0.05	0.53 ± 0.05	*p* = 0.036*
Down FR (a.u.)	0.20 ± 0.01	0.24 ± 0.01	*p* = 0.015*
Upward transition (°)	14 ± 2	10 ± 2	*p* = 0.167

Local cortical activity during Up states is synchronized at gamma frequencies [40, 41]. Synchronization at this frequency band has been related to cognitive processing [42, 43] and has been reported to be affected in mouse models of aging and mental disability [19, 22]. WBS mice express some of the cognitive deficits present in WBS patients [26], so we hypothesized that high-frequency content could be altered in these animals. To test this hypothesis, we quantified the high-frequency content in the gamma band (32–100 Hz) by computing Fourier analyses during Up states in WBS and WT mice. We found that the power of the gamma band was decreased in WBS mice compared with WT (Fig. 2). This result suggests that the cognitive impairment described in WBS mice [26] might, at least in part, be mediated by a deficit in the capacity of the frontal cortical network to synchronize at high frequencies, as is the case in a model of Down syndrome for example [22].

**Fig. 3:**
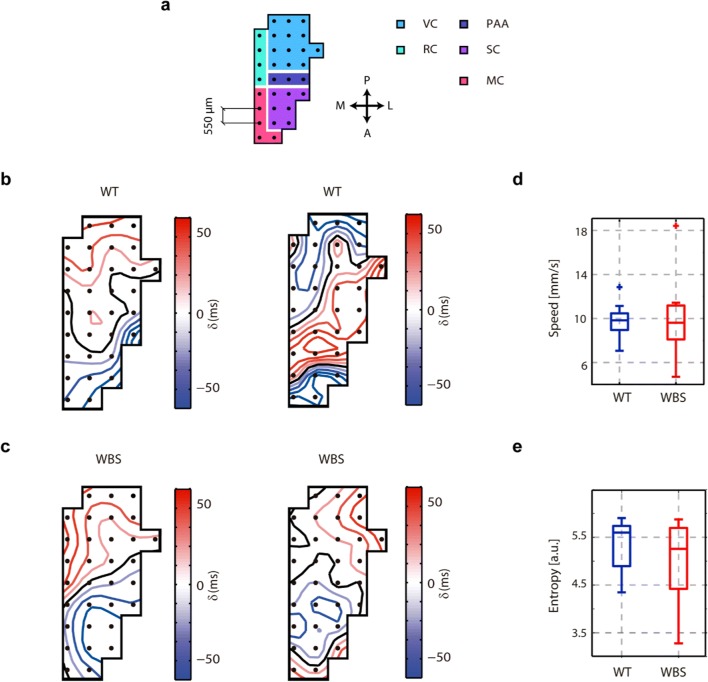
Slow-wave propagation in the cortical surface in WT and WBS mice. (**a**) Scheme of the superficial 32-channel electrode array. In colors, the areas covered by the array according to the stereotaxic coordinates (VC=visual cortex, RC=retrosplenial cortex, PAA=parietal association area, SC=somatosensory cortex, MC=motor cortex; P=posterior, A=anterior, L=lateral, M=medial) (**b**) Example of two spatiotemporal patterns of wave propagation in WT animals. The color scale represents the time delays in milliseconds. (**c**) Example of two spatiotemporal patterns of wave propagation in WBS animals, color code as in panel **b**. (**d**) Box plots of the average absolute speed of propagation across the experiment in WT and WBS animals. (**e**) Box plots of the entropy of the spatiotemporal patterns of the waves in WT and WBS animals. No differences were found between WT and WBS animals

**Fig. 4:**
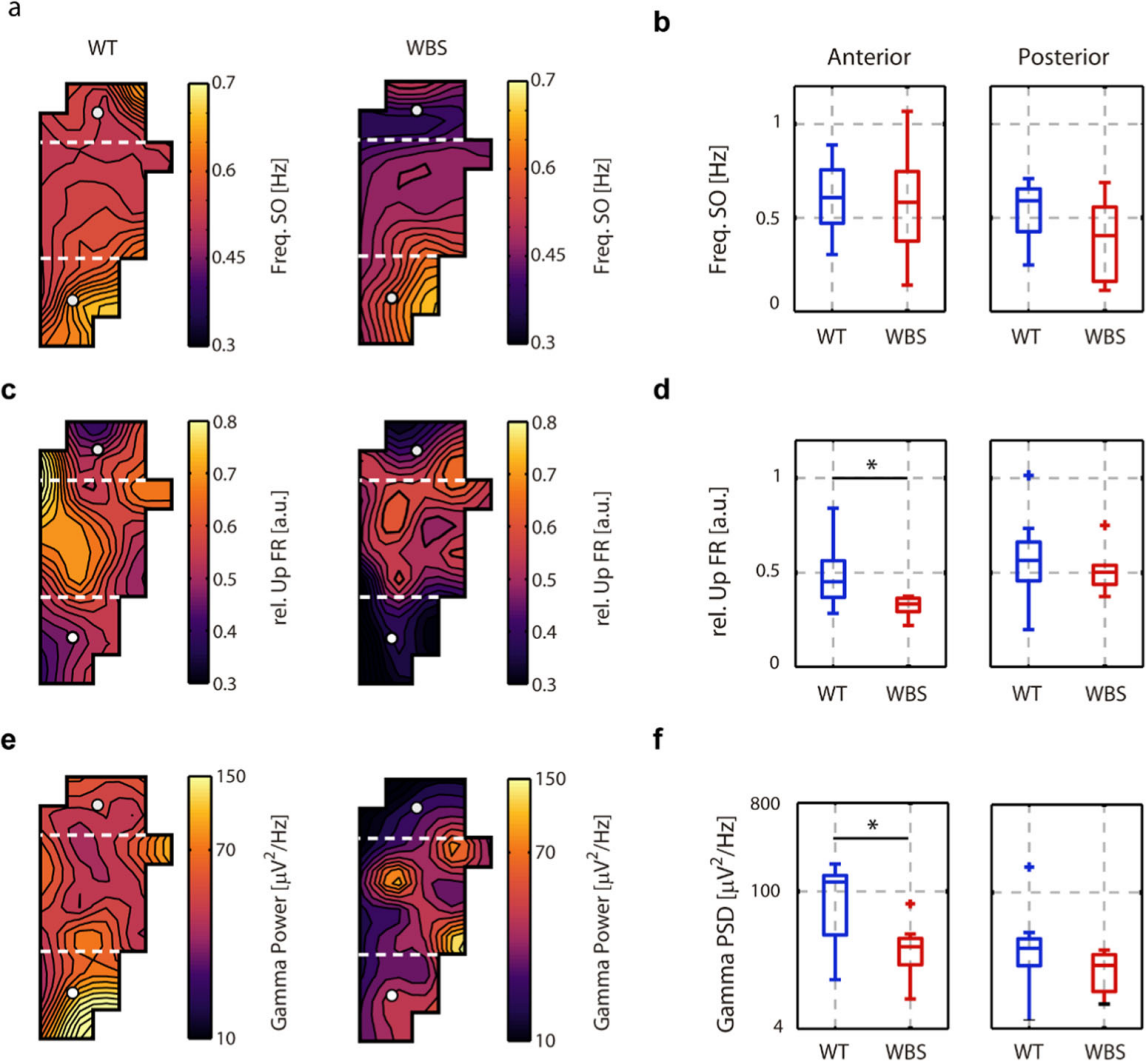
Superficial distribution of SO parameters in WT and WBS mice. (**a, c, e**) 2D interpolation of the SO frequency, FR of the Up state relative to the Down state, and gamma power, respectively, in the 32-channel array for the mean population of all WT (*N* = 9) and WBS (*N* = 8) mice. Dashed white lines delimit the anterior and posterior regions represented in the boxplot. (**b**) Mean frequency SO values for the anterior and posterior regions in all WT and WBS mice. No significant differences were found. (**d**) Box plots of the mean relative FR of the Up state for the anterior (*p* = 0.008, Mann–Whitney *U* test) and posterior regions. (**f**) Box plots of gamma power (32–100 Hz) for the anterior (*p* = 0.0015, Mann–Whitney *U* test) and posterior regions

In order to further explore the cortical dynamics in WBS mice, we used surface–subdural arrays of 32 electrodes (micro-ECoG) covering different areas (visual, retrospenial, parietal association, somatosensory, and motor cortex) of one hemisphere. We first measured the propagation of the slow waves [22, 29, 44], a property that is highly revealing of the underlying network. We quantified the diversity of the propagating waves as the entropy in the plane of the first and second principal components of the waves (Fig. 3). No differences were found in this parameter, illustrating that even when there is a deficit in excitability, the dynamics of wave propagation are still preserved, and therefore there is not a complete disruption of cortical activity. However, when we analyzed the parameters of the SO from the surface of the brain (Fig. 4), we found that the most frontal areas covered by the electrodes (Fig. 4b–f)—corresponding to somatosensory and motor areas—also displayed significant decreases in firing rates and gamma synchronization in WBS with respect to WT (Fig. 4d, f), while the frequency of the SO remains stable (Fig. 4b). This finding represents further evidence that the decreased firing rate and gamma synchronization is robust enough to be evident in a larger area (larger than the frontal association cortex), with a frontal lobe distribution, and different from those in the posterior areas of the brain (namely, the visual cortex, Fig. 4a–e).

**Fig. 5:**
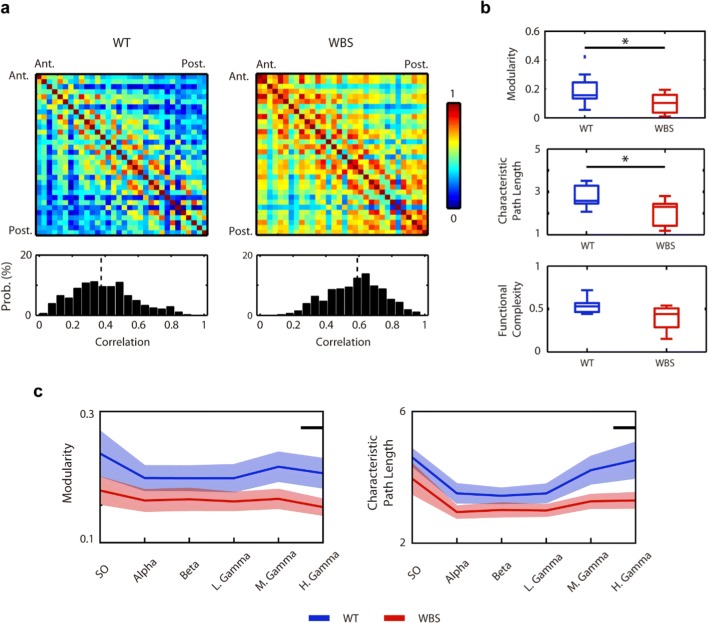
Analysis of functional connectivity in the WT and WBS mice. (**a**) Up, mean pairwise correlation matrices for the WT and WBS mice. Down, distribution of correlation values (*p* = 1.72·10^−43^, Kolmogorov–Smirnov). Vertical black dashed lines show the median value of the distributions**.** (**b**) From up to down, group differences in modularity (*p* = 0.036, Mann–Whitney *U* test), characteristic path length (*p* = 0.036, Mann–Whitney *U* test), and functional complexity. (**c**) Frequency-band filtered values of the modularity and characteristic path length (SO 0.1–1 Hz, alpha 8–12 Hz, beta 15–32 Hz, low gamma 32–50 Hz, middle gamma 50–70 Hz, high gamma 70–100 Hz). Black line indicates *p* value < 0.05, Mann–Whitney *U* test

Alterations in specific patterns of neuronal synchronization are characteristic of different neuropsychiatric and neurodevelopmental conditions [45, 46]. Indeed, previous studies in WBS patients have shown alterations in the overall level of cortical coherence [10, 11]. Thus, in order to test whether the alterations in synchronization we observed at the level of the frontal areas were a general phenomenon that could be affecting the integrity of the cortical network in our mouse model, we next studied the overall functional connectivity of the cortical network. Analyses based on the coherence of the raw micro-ECoG signal between all pairs of electrodes revealed an increase in the overall magnitude of functional connectivity in WBS mice in comparison with WT littermates (Fig. 5). This was especially apparent when plotting the distribution of the correlation values, indicating a bias towards a higher level of correlation in WBS mice (Fig. 5a).

Complex dynamic systems like the cerebral cortex require a balance between the level of integration and segregation [47]. Integration can be estimated by the level of coherence, while the level of segregation can be conceptualized as the capacity of the different functional elements of the cortical network to perform specialized functions [48]. Previous studies using murine models of mental disability have reported alterations in functional connectivity to be linked to abnormalities in the integration–segregation balance [49]. We thus wondered whether the higher correlation values observed in our mouse model of WBS could be related to alterations in the balance between segregation and integration of the cortical network. To answer this question, we calculated the degree of modularity (segregation) and path length (inverse of integration) in our population of WBS and WT mice based on the recorded micro-ECoG signals. As shown in Fig. 5b, WBS mice showed lower modularity levels and shorter path length as compared with WT, thus revealing that the overall level of complexity was affected in the cortical network of WBS mice.

We next considered whether this imbalance between segregation and integration in the cortical network of WBS mice was a non-specific phenomenon affecting general cortical dynamics or was otherwise restricted to a specific frequency band. To answer this, we calculated the degree of modularity and path length at different frequency bands, ranging from SO (0.1–1 Hz) to high gamma (70–100 Hz). Both modularity and path length were consistently lower in WBS mice compared with WT, although it was at the high-gamma frequency range where these differences became stronger (Fig. 5c). These results were in agreement with the abovementioned impairment in the gamma-range high-frequency component during Up states and are thus suggestive of a general alteration in the synchronization at gamma frequencies that may underlie the neurocognitive deficits present in WBS.

**Fig. 6:**
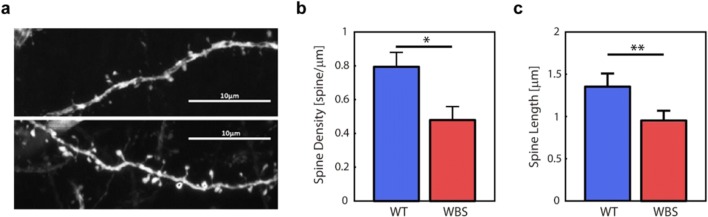
Dendritic spines in YFP+ pyramidal neurons in the frontal cortex. (**a**) Representative photographs of dendritic spines on apical proximal dendrites of frontal cortex pyramidal neurons in WBS (top) and WT (bottom) mice are shown. (**b**) A significant reduction (*p* = 0.015) in spine density was found in apical dendrites of WBS mice (*N* = 6 mice/genotype). (**c**) Spines in apical dendrites of WBS mice were significantly shorter (*p =* 0.005) when compared with WT mice (*N* = 518–689 spines, 6 mice/genotype). Data are presented as mean ± SEM. *p* values are shown with asterisks indicating values that they are significantly different (Mann–Whitney *U* test . *p < 0.05; **p < 0.01)

Dendritic spines have been shown to participate in the generation of a distributed connectivity matrix network with fan-out and fan-in interactions necessary for the appearance of linear functional integration capacities [50]. Moreover, dendritic spines are the main locus for excitatory synapses in cortical pyramidal neurons [51]. Interestingly, several neuropsychiatric conditions, including those within the autism spectrum, have been reported to express atypical morphologies and numbers of dendritic spines [52]. We thus queried whether the affected functional coherence and excitability present in our mouse model of WBS could be the consequence of an anatomical deregulation at the level of the synapse. To test this possibility, we studied the spine density of apical dendrites in YFP+ pyramidal neurons in the frontal cortex of 12 mice (WT = 6; WBS = 6). As shown in Fig. 6, we found that the spine density in apical dendrites was significantly reduced by 40% in WBS mice (WT, 0.793962 ± 0.03646; WBS, 0.479125 ± 0.03522; *p =* 0.015, Mann–Whitney *U* test) and that spines were significantly shorter (*p =* 0.005, Mann–Whitney *U* test) when compared with WT. These results thus offered a possible anatomical correlate to the physiological alterations already reported in our mouse model regarding complexity and excitability.

**Fig. 7:**
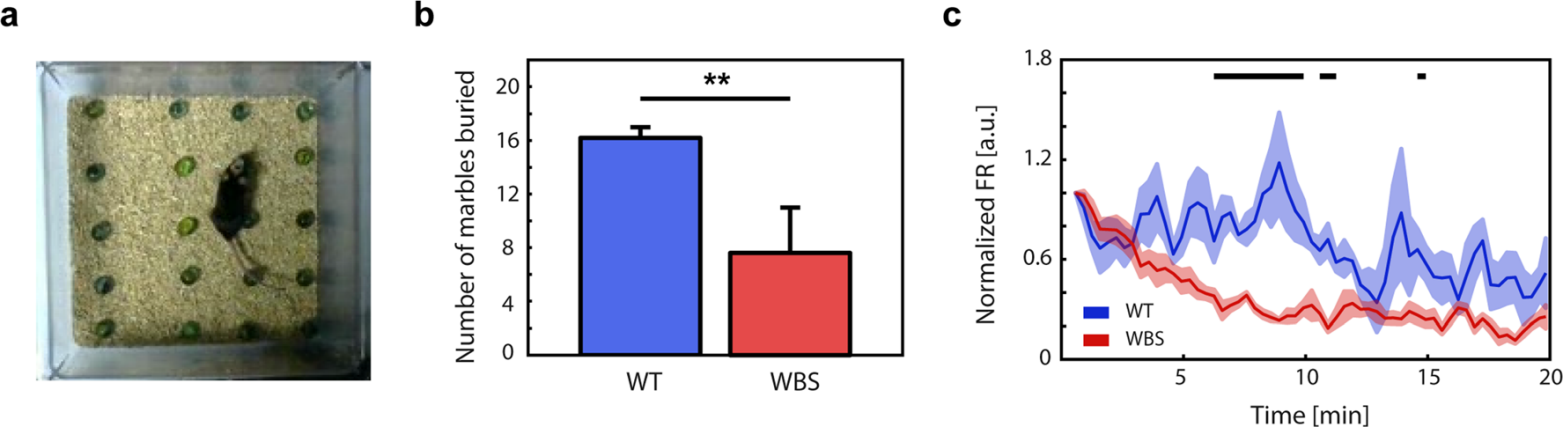
Cortical activity in the awake-behaving animal in WT and WBS mice. (**a**) Marble-burying task. Image taken at the beginning of the trial. (*N*_WT_ = 5, *N*_WBS_ = 5). (**b**) Performance of WT and WBS during the marble-burying test (*p* = 0.008, Mann–Whitney *U* test). (**c**) Grand average of the firing rate during the development of the task normalized with respect to that at the initiation of the task. The shade represents the standard error of the mean. The firing rate was larger in WT animals, and the significant windows are indicated above with a black line (*p* < 0.05)

Finally, since our previous measurements had been done in the anesthetized mice, we recorded from the frontal association cortex of awake-behaving mice. We chose for this a marble-burying task (see “Methods”) (Fig. 7a). The marble-burying test is a task known to be affected by anxiety-like behavior [53], and anxiety is a psychiatric disorder prevalent in most (more than 80%) individuals affected by WBS [54, 55]. We investigated the performance of the task and found that WBS mice performed significantly worse than WT mice (Fig. 7b), burying about half the marbles. Furthermore, the cortical recordings during the development of the task revealed a significantly lower population firing rate in WBS (Fig. 7c, grand average) during the performance of the task. Even when we cannot propose any cause–effect between the firing rate in the frontal cortex and task performance, this data is a valuable illustration that the decreased firing rates observed in the Up states in WBS are indeed persistent in the awake-behaving animal, probably reflecting a decreased synaptic connectivity as suggested by the reduced number in dendritic spines.

## Discussion

In this study, we characterized the properties of the cortical network in a murine model of WBS in an effort to understand some of the micro- and mesocortical functional characteristics underlying the cognitive and behavioral phenotype of WBS. Our study concentrated—although not exclusively—on the frontal cortex. We chose this area because it subserves higher-order cognitive functions and social behavior, functions that are altered in WBS [1]. We presented results regarding the anatomy of this cortical area in both WBS and WT mice, finding that while the macroproperties such as the cortical layers are well preserved, microaspects such as dendritic spines are reduced in WBS. This reduction of dendritic spines has important functional implications, and it is precisely these functional aspects that we focused on, in particular, on the functional emergent properties of cortical circuits, resulting from the analysis of slow oscillatory rhythms. SO are a highly synchronized activity generated by the cerebral cortex network during slow-wave sleep and deep anesthesia, which has been proposed as the default activity of the cortex [56, 57]. This spontaneous activity is highly informative of the state of the underlying circuits, and to explore these circuits, we quantified different parameters of the SO. By doing so, we found a reduced synchronization in the gamma-band frequency and reduced population firing rates of neurons in WBS. The decreased population firing rates under anesthesia persisted in the awake-behaving WBS mice, suggesting a diminished network excitability that could be associated with the decreased synaptic connectivity suggested by the decreased number of dendritic spines in the area. A deficit in myelination in the frontal cortex reported in WBS, and thus, a decrease in effective axonal transmission [58] could also be a contributing factor to the decreased network excitability. Finally, we explored the dynamics of the larger cortical network, observing a significantly reduced modularity of the network, although not enough to decrease its functional complexity [31].

We demonstrated that in the frontal cortex of WBS, SO are more variable and the firing rates in Up states are lower. Interestingly, the duration of Up states have been reported to be inversely related to the firing rates in the local population [59, 60]. What could be the mechanisms underlying these changes? A decreased excitability in the cortical circuits in frontal areas would generate lower firing rates and thus elongated and more irregular Up states and oscillatory cycles. Our data support this hypothesis as the average firing rate during Up states was reduced in the cortex of WBS mice as was gamma synchronization. Previous studies in the cortex of different murine models of cognitive disability such as Down syndrome [22] have also reported a decreased level of cortical network excitability. A suggested mechanism for this is the fact that the termination of Up states is determined by the opening of activity-dependent hyperpolarizing conductances mediated by Ca^+2^- and Na^+^-dependent K^+^ channels [60–62]. It is therefore conceivable that if the elements of the local cortical network are less excitable and have lower firing rate levels, the intracellular accumulation of Ca^+2^ and Na^+^ would be less, thus altering the repolarization mechanisms and elongating Up states. However, why would excitability and therefore, firing rates, be decreased in WBS? Dendritic spines are the main locus of excitatory synapses derived from glutamatergic axons on cortical pyramidal cells [51]. Alterations in dendritic spines are often found in neuropsychiatric disorders (for a review, see [63]). The number and morphology of these spines has been reported to be anomalous in several autism-related disorders [52], where the level of excitability and overall cortical dynamics are also affected [20, 22]. We have shown here that the number and length of dendritic spines are reduced in our mouse model of WBS, which suggests less excitatory recurrency in the circuit and lower firing rates. Indeed, at the level of the frontal cortex, we found lower densities of dendritic spines. The reduced spine density present in our mouse model would imply a reduced synaptic connectivity and an effect on the convergence of excitatory inputs into cortical pyramidal neurons [51], which would affect the capacity of the cortical network to linearly integrate excitatory inputs in a non-saturating manner [50]. In this regard, we report that the cortical network of our WBS mouse model expresses high functional connectivity alongside abnormally high integration capacity (lower path length) and low segregation levels (modularity); alterations that are especially notorious at gamma-like frequency bands. These results are in agreement with previous findings in humans reporting increased overall cortical connectivity [11] and suggest that the cognitive deficits previously reported in this murine model of WBS [26] may be associated with the interruption of the equilibrium between integration and segregation at gamma frequencies. The abovementioned alterations in the morphology and number of dendritic spines at the level of the frontal cortex might explain, at least partially, the origin of these functional abnormalities, which may hamper the capacity of the cortical network to distribute and integrate information in order to execute specialized functions. Interestingly, we observed decreased excitability during SO, but this feature persists in the awake, task-performing animal, enhancing the evidence that it is a feature intrinsic to this pathology.

Even when our results show that the number and length of dendritic spines in pyramidal neurons of frontal cortex were decreased, other authors (Chailangkarn et al. [64]) have indeed reported an increase in the number and length of dendritic spines in humans. We could attribute some of the differences to the different areas (frontal versus S1, M1, V2), different species (human vs mouse), or to the fact that we concentrated on apical dendrites while Chailangkarn et al. do not specify this*.* However, it seems relevant that our physiological data is congruent with the anatomical observation of a decreased number and length of dendritic spines that would imply reduced connectivity and therefore lower excitability and population firing rates.

The observation that neither the duration of the Up and Down states nor the frequency of the SO, nor even the slow-wave propagation (which results from the integration of different features of the local and distant network) were affected in WBS suggests that the functional alterations are not massive. Indeed, even when there are specific alterations, there is additionally maintenance of several anatomical and functional properties, suggesting a limited alteration and maybe a successful compensation via network homeostatic mechanisms. This is coherent with the cognitive disabilities in this syndrome, which are also moderate. One example of disability in this mouse is the poor performance in the marble-burying test. Even if the decreased frontal network excitability could contribute to decreased performance reflected in the marble-burying test, we cannot rule out reduced motor activity as an additional mechanism, as has been described by Barak et al. [58]. Indeed, we also observed decreased activity in sensorimotor areas (S1 and M1 and M2).

Regarding the irregularity of the waves, also associated with decreased excitability [65], the frontal cortex has been posited to be the origin of slow-wave initiation and expresses highly regular slow waves both in mice and humans [15, 66, 67]. Thus, the presence of more irregular slow waves in the frontal cortex of WBS mice suggests an alteration in the underlying recurrent dynamics of the cortex, possibly related to a displaced excitatory–inhibitory balance. This feature has already been described in other mouse models of autism [20]. Three autistic behavioral domains are impaired in WBS (social interaction, communication, stereotypies) [68]. Indeed, WBS patients can exhibit restricted and stereotyped behaviors or interests, such as hypersensitivity to sounds, picky eating, inflexibility, ritualism, and obsessive and perseverating attitudes [69]. Because SO are also the dominant pattern during slow-wave sleep, a relationship may exist with the altered sleep patterns in WBS in humans [7, 8].

In sum, our data show that the cortical network of our WBS mouse model is both functionally and cytoarchitectonically affected. Anomalous frontal cortex pyramidal cell dendrite density and morphology, alongside affected integration and segregation capacity in conjunction with abnormal functional connectivity patterns, especially in the gamma-like frequency range, were described. These alterations may hinder the complexity of the cortical network and may be translated into the neurocognitive deficits expressed in WBS.
